# Supplementary material for: Finger stick blood test to assess postvaccination SARS‐CoV‐2 neutralizing antibody response against variants
Source: Bioeng Transl Med. 2022 Feb 10;7(2):e10293. doi: 10.1002/btm2.10293 (PMC9115707; doi:10.1002/btm2.10293)
Supplement: Supplementary file 1 — Appendix S1: Supporting Information [file BTM2-7-e10293-s001.docx]

**Finger stick blood test to assess post vaccination SARS-CoV-2 neutralizing antibody response against variants**

Sing Mei Lim^1,†^ , Hoi Lok Cheng^1,†^ Huan Jia^1,†^, Patthara Kongsuphol ^1, †, ‡^, Bhuvaneshwari D/O Shunmuganathan ^2^, Ming Wei Chen^3^, Say Yong Ng^1^, Xiaohong Gao^3^, Shuvan Prashant Turaga^4^, Sascha P. Heussler^4^, Jyoti Somani ^5^, Sharmila Sengupta ^5^, Dousabel MY Tay ^6^, Megan E. McBee^1^, Barnaby E. Young ^7,8,9^, Paul A. MacAry^2,10^, Hadley D. Sikes^1,6,*^, Peter R. Preiser^1,6,*^

**Authors’ Affiliations:**

^1^ Antimicrobial Resistance Interdisciplinary Research Group (AMR-IRG), Singapore-MIT Alliance in Research and Technology (SMART); #03-10/11 Innovation Wing, 1 CREATE Way, Singapore 138602.

^2^ Department of Microbiology and Immunology, Yong Loo Lin School of Medicine, National University of Singapore (NUS); 5 Science Drive 2, Blk MD4, Level 3, Singapore 117545.

^3^ School of Biological Sciences (SBS), Nanyang Technological University (NTU); 60 Nanyang Dr, Singapore 637551.

^4^ Attonics System Pte. Ltd. 10 Anson Road, #12-01 International Plaza, Singapore 079903.

^5^ National University Hospital (NUH); 5 Lower Kent Ridge Rd, Singapore 119074.

^6^ Department of Chemical Engineering, Massachusetts Institute of Technology (MIT); 25 Ames Street, Building 66 Cambridge, MA 02139, USA.

^7^ National Centre for Infectious Diseases (NCID); 16 Jalan Tan Tock Seng, Singapore 308442.

^8^ Department of Infectious Diseases, Tan Tock Seng Hospital; 16 Jalan Tan Tock Seng, Singapore 308442.

^9^ Lee Kong Chian School of Medicine; 59 Nanyang Drive Singapore 636921.

^10^ Life Sciences Institute (LSI), National University of Singapore (NUS); #05-02, 28 Medical Drive, Singapore 117456.

^†^ SM, HC, HJ and PK contribute equally as co-first authors.

^‡^ PK current affiliation is Thrixen Pte. Ltd.

* HDS and PRP are co-corresponding authors. Emails: [sikes@mit.edu](mailto:sikes@mit.edu); [prpreiser@ntu.edu.sg](mailto:prpreiser@ntu.edu.sg).

**Supplementary Information**


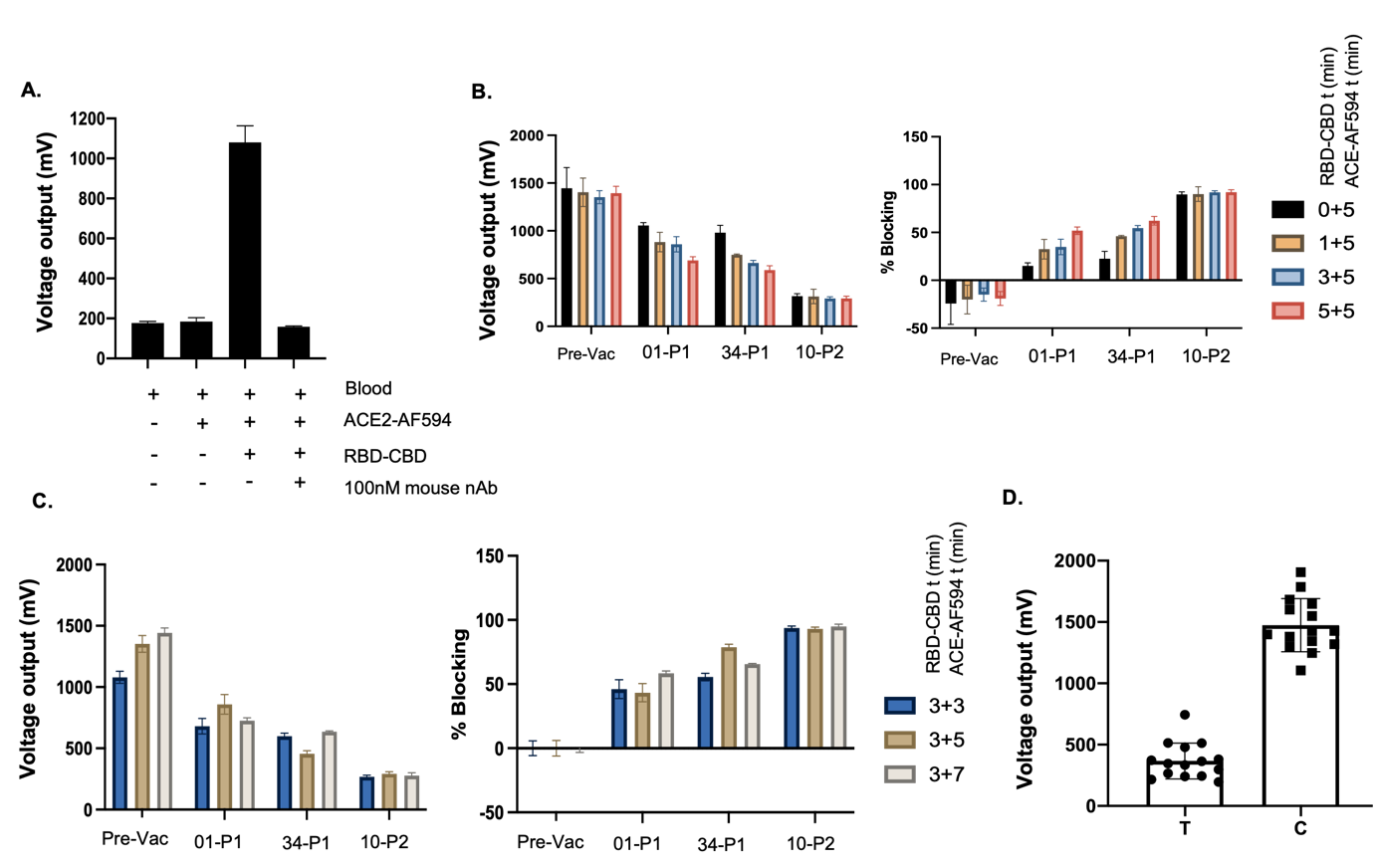


**Fig. S1. Optimization of modified cpVNT with Alexa Fluor594 labeled ACE2.**

**A.** Fluorescence intensity (voltage output) measured with blood samples in the presence and absence of mouse monoclonal SARS-CoV-2 nAb. **B.** Four different participants’ samples: pre-vaccination (Pre-Vac), post 1^st^ dose (01-P1, 34-P1) and post 2^nd^ dose (P2) were used for optimization of RBD-CBD incubation time and **C.** ACE2-AF594 incubation time. All samples were tested in triplicates. **D**. Comparison of fluorescent intensity between Test spot-T and Control spot-C with 15 post 2nd dose vaccinees’ whole blood samples.


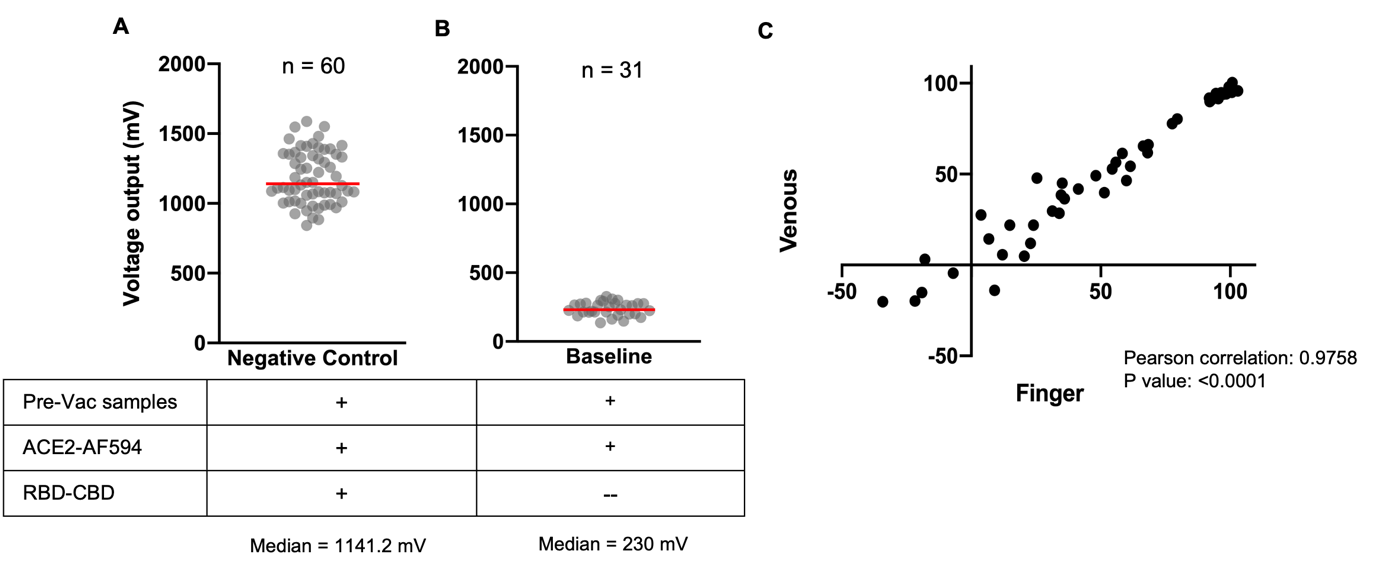


**Fig. S2. Signal intensity for negative control and baseline as well as correlation of plasma and whole blood samples with modified cpVNT.**

**A**. Fluorescence intensity (voltage output) measured from 60 pre-vaccination blood samples, red line represents median of the distribution, 1141 mV; which is the value used as NC for % blocking calculation. **B**. Total 31 samples (pre and post vaccination) were measured in the absence of RBD-CBD, where the red line is the median, 230 mV. **C.** A total of 46 matching samples of finger stick and venous blood showed strong correlation (Pearson r: 0.9758, p< 0.0001) in percent blocking detection using the modified cpVNT.


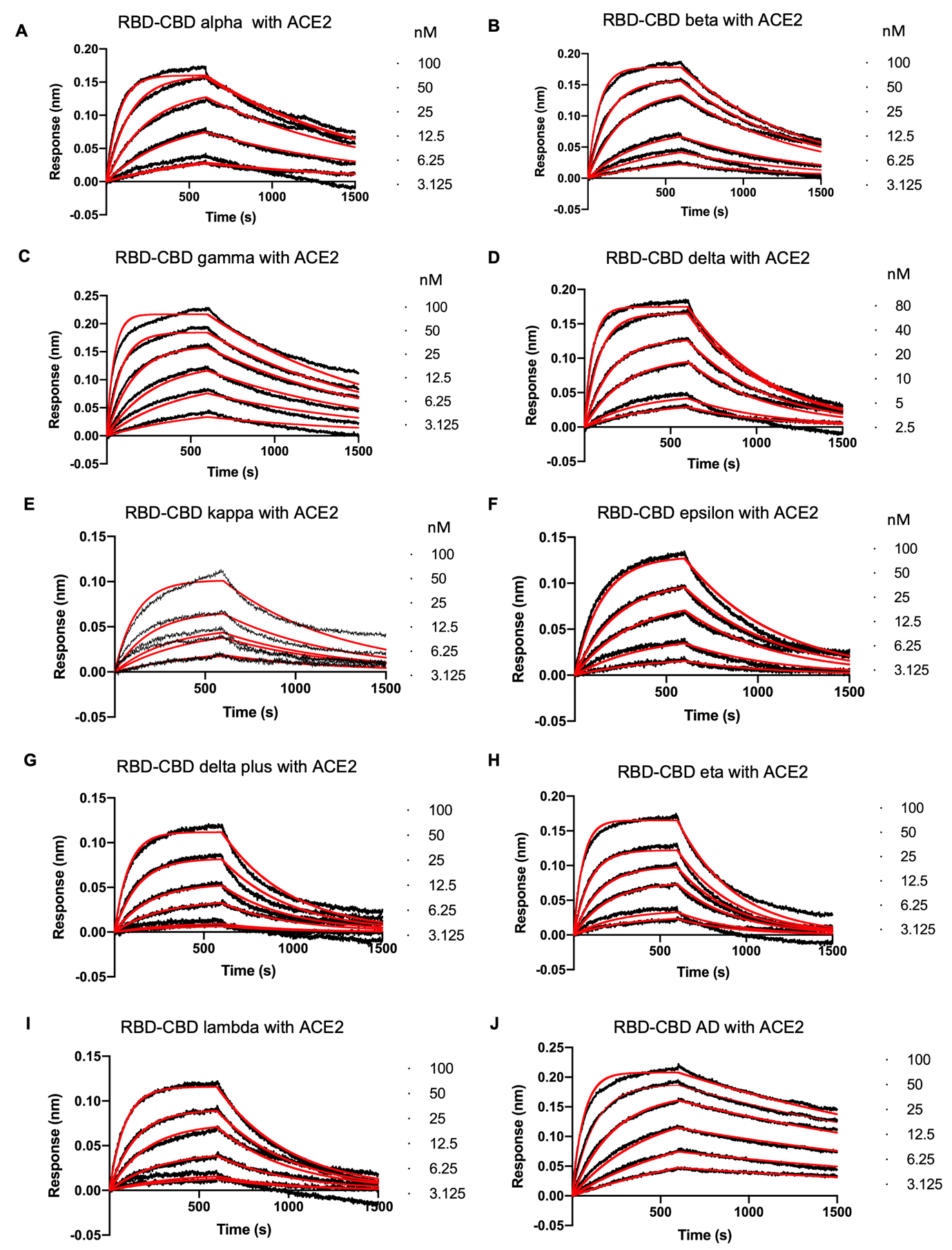


**Fig. S3 Biolayer interferometry profiles for RBD-CBD variants with biotinylated ACE2 immobilized on streptavidin biosensors.**

**A.** RBD-CBD alpha, **B**. RBD-CBD beta, **C**. RBD-CBD gamma, **D**. RBD-CBD delta, **E**. RBD-CBD kappa, **F.** RBD-CBD epsilon, **G.** RBD-CBD delta plus, **H.** RBD-CBD eta, **I.** RBD-CBD lambda and **J.** RBD-CBD AD


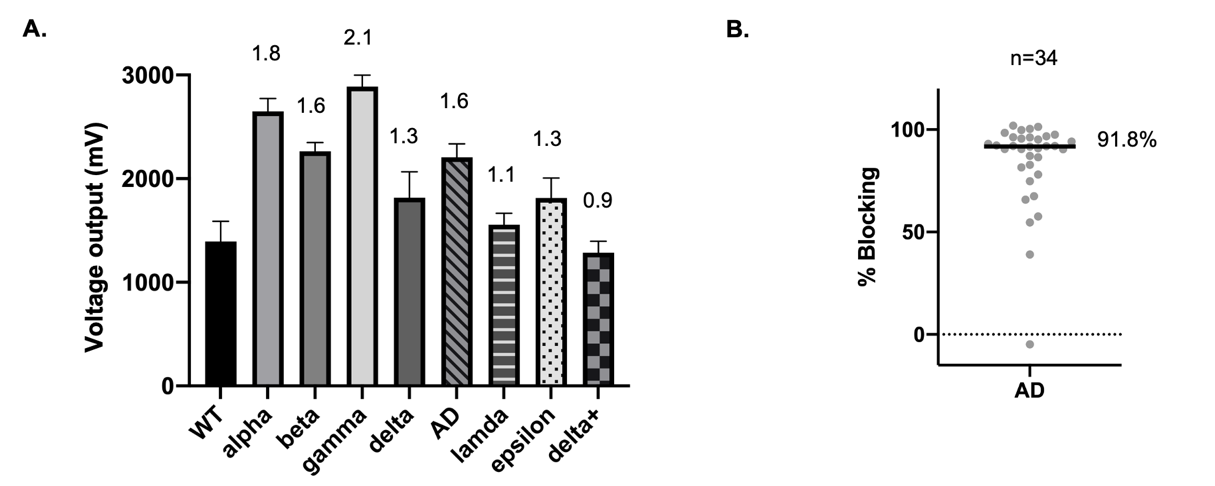


**Fig. S4 Signal intensity of RBD-CBD variants’ response with fluorescence labelled ACE2.**

**A**. The fluorescence intensity (voltage output) of pre vaccination blood sample when tested against wildtype (WT) and RBD-CBD variants of different binding affinity against ACE2. The numbers above each bar indicate fold of increase in intensity as compared to WT. **B.** The nAb percent blocking measured from 34 post 2^nd^ dose vaccinated samples (within 3 months) against AD, the hypothetical RBD-CBD.

**Table S1. Fluorescence intensity (voltage output) measured from two batches of ACE2-AF594 with pre vaccinate blood samples for negative control calibration.**

| ACE2-AF594  Batch | RBD-CBD Concentration  (nM) | Fluorescence intensity for NC | # Samples | Fluorescence intensity for baseline | # Samples |
| --- | --- | --- | --- | --- | --- |
| 1 | 10 | 1088.7 (±179.8) | 17 | 181.1 (±18.3) | 7 |
| 2 | 10 | 1235.5 (±185.9) | 43 | 254.1 (±25.9) | 24 |

**Table S2: Samples used for modified cpVNT-blood in comparison with pVNT, cPass and cpVNT-plasma**

| Sample ID | Vaccination status | % Neutralization in pVNT | % Inhibition in cPass | % Blocking cpVNT-blood | % Blocking cpVNT-plasma |
| --- | --- | --- | --- | --- | --- |
| 0001-V-V05-PV | Pre-Vac |  | -7.763312 | 22.94239 |  |
| 0003-F-V03-PV | Pre-Vac |  | -17.49623 | -18.20988 |  |
| 0004-V-V01-PV | Pre-Vac | 0 |  | 19.75356 | 19.01346 |
| 0004-V-V04-PV | Pre-Vac |  | -2.99429 | -7.40E-15 |  |
| 0004-F-V06-PV | Pre-Vac |  | -5.805046 | 12.53561 |  |
| 0005-V-V01-PV | Pre-Vac |  | -13.7654 | 27.02332 |  |
| 0007-V-V01-PV | Pre-Vac | 0 | -10.4598 | 11.35117 | 11.89958 |
| 0008-V-V01-PV | Pre-Vac | 0 | -14.8892 | 11.96845 | -21.78846 |
| 0009-V-V01-PV | Pre-Vac |  | -8.86967 | 13.20597 |  |
| 0011-V-V01-PV | Pre-Vac |  | -11.27776 | -27.88066 |  |
| 0013-F-V06-PV | Pre-Vac |  | -5.521211 | -12.65432 |  |
| 0013-F-V08-PV | Pre-Vac | 0 |  | 8.40192 |  |
| 0022-F-V01-PV | Pre-Vac |  | 1.500837 | -11.34092 |  |
| 0022-V-V01-PV | Pre-Vac | 0.12 | 11.52455 | 21.31724 | 15.95626 |
| 0026-F-V01-PV | Pre-Vac |  | -6.576852 | -38.8203 |  |
| 0027-F-V01-PV | Pre-Vac |  | 3.549902 | 12.41427 |  |
| 0030-F-V01-PV | Pre-Vac |  | 7.101754 | 14.3 |  |
| 0036-V-V01-PV | Pre-Vac | 0 |  | 9.84225 | 11.50885 |
| 0042-V-V01-PV | Pre-Vac |  |  | 12.89438 | 8.254454 |
| 0054V-V01-PV | Pre-Vac |  |  | 1.646091 | -4.238932 |
| 0001-F-V09-P1-02W-MD | P1 |  | 66.0465 | 31.893 |  |
| 0001-V-V12-P1-05W-MD | P1 | 43.56 | 72.68333 | 36.07682 | 73.514022 |
| 0003-V-V06-P1-03W-PF | P1 | 1.03 | 44.83542 | 21.46776 | 17.1909 |
| 0004-V-V10-P1-03W-MD | P1 |  | 75.51875 | 22.39369 |  |
| 0009-V-V02-P1-03W-PF | P1 | 3.21 | 10.31425 | 28.45651 | 8.348457 |
| 0010-V-V03-P1-01W-MD | P1 |  | -8.83867 | 22.97668 | 19.5426 |
| 0010-F-V06-P1-03W-MD | P1 |  | 89.66134 | 73.07956 |  |
| 0011-V-V02-P1-03W-PF | P1 |  | 31.8281 | 34.12209 |  |
| 0012-V-V01-P1-03W-PF | P1 | 3.02 | 41.51574 | 39.16324 | 28.42025 |
| 0013-F-V09-P1-02W-MD | P1 |  | 67.62917 | 20.78189 |  |
| 0019-V-V2-P1-03W-PF | P1 | 37.75 | 63.88273 | 27.02332 | 50.4968 |
| 0020-F-V01-P1-04W-PF | P1 |  | 81.96858 | 31.48148 |  |
| 0022-F-V02-P1-01W-PF | P1 |  | 1.500837 | 10.90535 |  |
| 0022-V-V04-P1-06W-PF | P1 | 37.75 | 71.37292 | 41.46091 | 60.19754 |
| 0025V-V03-P1-6W-PF | P1 |  |  | 48.76543 | 53.05427 |
| 0045V-V05-P1-5W-PF | P1 |  |  | 61.21399 | 64.01905 |
| 0046V-V01-P1-3W-MD | P1 |  |  | 80.55843 | 96.97219 |
| 0049-V-V01-P1-03W-MD | P1 |  | 79.53542 | 39.47188 | 52.93668 |
| 0052-V-V01-P1-05W-PF | P1 | 36.48 | 72.69792 | 28.01783 | 54.58287 |
| 0053V-V01-P1-5W-PF | P1 | 44.46 | 71.42917 | 38.13443 | 74.68987 |
| 0054-V-V02-P1-03W-PF | P1 |  |  | 43.34705 | 33.47639 |
| 0062V-V01-P1-5W-MD | P1 |  |  | 75.5144 | 83.94967 |
| 0001-V-V16-P2-02W-MD | P2 |  | 96.88125 | 95.81619 | 99.88242 |
| 0002-V-V07-P2-07W-PF | P2 |  | 97.30353 | 104.3553 |  |
| 0002-V-V09-P2-14W-PF | P2 | 96.19 | 96.91042 | 90.84362 | 99.58845 |
| 0004-F-V12-P2-01W-MD | P2 |  | 71.09583 | 40.9808 |  |
| 0004-V-V14-P2-02W-MD | P2 | 97.46 | 96.775 | 91.80384 | 99.94121 |
| 0006-V-V01-P2-01W-MD | P2 | 48.63 | 91.3985 | 90.18098 |  |
| 0007-V-V08-P2-03W-MD | P2 | 94.56 | 96.81875 | 91.02304 | 95.18118 |
| 0009-V-V03-P2-03W-PF | P2 | 63.7 | 93.34792 | 84.22497 |  |
| 0009-V-V04-P2-12W-PF | P2 |  |  | 56.15305 | 63.6075 |
| 0010-F-V07-P2-01W-MD | P2 |  | 94.50406 | 88.40878 |  |
| 0024-F-V01-P2-08W-PF | P2 |  | 96.54536 | 85.69959 |  |
| 0028-F-V01-P2-09W-PF | P2 |  | 92.88269 | 81.44719 |  |
| 0029-F-V01-P2-05W-PF | P2 |  | 81.82083 | 63.3059 |  |
| 0034-V-V03-P2-02W-MD | P2 | 99.64 | 97.06042 | 96.46776 | 99.88242 |
| 0046V-V02-P2-3W-MD | P2 |  |  | 94.20439 | 95.70815 |
| 0067V-V01-P2-3W-PF | P2 |  |  | 79.13328 | 61.02064 |
| **Total samples** |  | **20** | **45** | **58** | **30** |

**Table S3: The percent inhibition of WHO International Standard and Reference Panel’s plasma measured using cPass^TM^ .**

| **WHO Plasma Standard & References** | **Neutralizing activity (IU/mL)** | **% Inhibition measured in cPass** |
| --- | --- | --- |
| International Standard | 1000 | 94.3 (±0.26) |
| International Standard (1:1 dilution) | 500 | 90.6 (±0.73) |
| Mid titre | 210 | 78.7 (±0.65) |
| Low titre | 44 | 19.0 (±3.2) |
| Low titre (1:1 dilution) | 22 | 8.3 (±1.4) |
